# Avoidable errors in deposited macromolecular structures: an impediment to efficient data mining

**DOI:** 10.1107/S2052252514005442

**Published:** 2014-04-14

**Authors:** Zbigniew Dauter, Alexander Wlodawer, Wladek Minor, Mariusz Jaskolski, Bernhard Rupp

**Affiliations:** a Synchrotron Radiation Research Section, Macromolecular Crystallography Laboratory, NCI, Argonne National Laboratory, Argonne, IL 60439, USA; b Protein Structure Section, Macromolecular Crystallography Laboratory, NCI at Frederick, Frederick, MD 21702, USA; c Department of Molecular Physiology and Biological Physics, University of Virginia, Charlottesville, VA 22908, USA; d Midwest Center for Structural Genomics, USA; e New York Structural Genomics Consortium, USA; f Center for Structural Genomics of Infectious Diseases, USA; g Enzyme Function Initiative, USA; h Department of Crystallography, Faculty of Chemistry, A. Mickiewicz University, Poznan, Poland; i Center for Biocrystallographic Research, Institute of Bioorganic Chemistry, Polish Academy of Sciences, Poznan, Poland; j k.-k. Hofkristallamt, 991 Audrey Place, Vista, CA 92084, USA; k Department of Genetic Epidemiology, Innsbruck Medical University, Schöpfstrasse 41, A-6020 Innsbruck, Austria

**Keywords:** macromolecular crystallography, model validation, Protein Data Bank

## Abstract

Whereas the vast majority of the more than 85 000 crystal structures of macromolecules currently deposited in the Protein Data Bank are of high quality, some suffer from a variety of imperfections. Although this fact has been pointed out in the past, it is still worth periodic updates so that the metadata obtained by global analysis of the available crystal structures, as well as the utilization of the individual structures for tasks such as drug design, should be based on only the most reliable data. Here, selected abnormal deposited structures have been analysed based on the Bayesian reasoning that the correctness of a model must be judged against both the primary evidence as well as prior knowledge. These structures, as well as information gained from the corresponding publications (if available), have emphasized some of the most prevalent types of common problems. The errors are often perfect illustrations of the nature of human cognition, which is frequently influenced by preconceptions that may lead to fanciful results in the absence of proper validation. Common errors can be traced to negligence and a lack of rigorous verification of the models against electron density, creation of non-parsimonious models, generation of improbable numbers, application of incorrect symmetry, illogical presentation of the results, or violation of the rules of chemistry and physics. Paying more attention to such problems, not only in the final validation stages but during the structure-determination process as well, is necessary not only in order to maintain the highest possible quality of the structural repositories and databases but most of all to provide a solid basis for subsequent studies, including large-scale data-mining projects. For many scientists PDB deposition is a rather infrequent event, so the need for proper training and supervision is emphasized, as well as the need for constant alertness of reason and critical judgment as absolutely necessary safeguarding measures against such problems. Ways of identifying more problematic structures are suggested so that their users may be properly alerted to their possible shortcomings.

## Abstract 2

The dual role of the Protein Data Bank as a repository of all macromolecular structures and as the major source of structural metadata for further analysis is discussed and suggestions are made on how to identify models that contain errors and could potentially degrade the quality of meta analyses.

## 1. Introduction   

Macromolecular crystallography (MX) is a highly interdisciplinary branch of science. Different aspects of conducting crystallographic experiments may require, in addition to the obvious need of knowledge of crystallography itself, also being versed in mathematics, physics, chemistry, biology and medicine (Ewald, 1948 ▶). In addition, modern crystallography involves very extensive use of computational capabilities. In spite of the enormous progress in the theory and practice of MX in recent years, and the availability of highly automated and user-friendly hardware and software tools, MX is far from trivial. Proper design of experiments and the interpretation of MX data still require serious engagement of the human brain.

Despite significant validation and quality-control efforts by the main repository for macromolecular structure models, the Protein Data Bank (PDB; Berman *et al.*, 2000 ▶, 2003 ▶; Dutta *et al.*, 2008 ▶; Read *et al.*, 2011 ▶), some of the models still contain errors of various kinds. Presented here are typical cases of errors or inconsistencies originating from different sources selected from the PDB or from available publications. The examples illustrate different types of problems that are not uncommon, and yet may seriously impede follow-up studies and data-mining efforts. Discussion of how to avoid them is necessarily brief here; a fuller treatment can be found, for example, in our recent review (Wlodawer *et al.*, 2013 ▶) and textbook (Rupp, 2009 ▶). It is not our intent to offend or ridicule the colleagues whose structures have been used as illustrations and, in fact, we have also included examples of our own provenance. The principal purpose is to remind the MX community that proper validation of all aspects of crystallo­graphic models is not only prudent and necessary practice, but is also critical for follow-up studies. Lack of attention to detail may ultimately lead to embarrassing and regrettable results. The presence of (largely preventable) errors in individual structures can mislead research that depends on the information that they contain, such as structure-guided drug design, in addition to the introduction of outliers into metadata obtained in the course of global analyses of the available crystal structure models. The users of the PDB, especially those who are not expert crystallographers themselves, can benefit from a clear identification of model entries that are questionable owing to mistakes in formal book-keeping, owing to errors resulting from deficient modeling, or by representing test cases from methodological development. In addition, when a number of structures are part of a series of closely related models, it might be useful to identify the most representative models. This is not a straightforward task, as the criteria that are used to select a representative search model for molecular replacement may differ from those to select a most appropriate structure for drug-discovery studies.

## 2. Reasoning and validation   

As a discipline concerned with the interpretation of experimental data, MX follows the scientific epistemology of empirical science based on the well established and universally accepted concepts of inductive reasoning, which were developed during the age of the Enlightenment in the 17th to 18th centuries. In the context of the validation of macromolecular structures (which are only atomic models of the actual molecules, based on the interpretation of the primary evidence in the form of electron density derived from diffraction data) two specific thoughts will guide us and thus deserve special attention.

Early on in the incipient phases of the Enlightenment, Sir Francis Bacon in his work *Novum Organum Scientiarum* notes that

the human understanding is not composed of dry light, but is subject to influence from the will and the emotions, a fact that creates fanciful knowledge; man prefers to believe what he wants to be true…for what man had rather were true he more readily believes

(Bacon, 1620 ▶). Bacon’s statement reminds us that wishful thinking and self-deception in the course of electron-density interpretation are, next to poor training and negligence, perhaps the most common reasons for the creation of flawed structure models. Such human folly is indeed known in sociology as expectation and confirmation bias (Koehler, 1993 ▶).

A concept evolved during a later period of the Enlightenment by The Reverend Thomas Bayes (a fellow of the newly established Royal Society, whose members essentially formed the basis of scientific epistemology as practiced today) deserves particular attention in the context of model validation. Published posthumously (Bayes, 1763 ▶), Bayes’ theorem places inductive reasoning in a framework of formal logic and simply states that the posterior model likelihood is proportional to the data likelihood times the prior probability of the model being correct independently of the actual data. Expressed in terms of data and model as a joint conditional probability (*P*), Bayes’ theorem in its simplest form reads
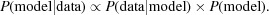



Although details regarding the application of Bayesian concepts to validation have been discussed elsewhere (Rupp, 2009 ▶, 2010 ▶; Pozharski *et al.*, 2013 ▶), one can immediately realize that in model validation guided by Bayesian reasoning, two terms need to be examined to estimate the likelihood of a model being correct: (i) how well the model reproduces the primary evidence (*e.g.* its fit or correlation to the electron density) and (ii) how well the model complies with independently acquired prior knowledge about its intrinsic properties (primarily reasonable stereochemistry, but all other known laws of nature as well). The important point here is that generally both terms together are necessary for complete evaluation: the fit against electron density alone is almost never precise enough to ensure proper stereochemistry, whereas evaluation of the stereochemistry alone does not inform us whether the model fits the local density. It is the combination of both terms that decides whether one is dealing with an error, an exciting feature, or just a lack of evidence. It is in fact often the point of disagreement between hard evidence and prior expectations where interesting discoveries are made and scientific revolutions emerge (Kuhn, 1970 ▶).

### 2.1. Classes of errors and inconsistencies   

#### 2.1.1. Inconsistent data presentation in PDB files   

One class of errors that can be readily detected by gross discrepancy with prior expectations involves nuisance errors, such as wrong data entries in the PDB header records, or in the data-collection and refinement statistics tables in publications. Such errors are also the easiest to intercept on deposition (although they are not necessarily easy to correct if the author of the entry does not respond to warnings in a PDB validation report). Examples are given in §§[Sec sec3.2]3.2, [Sec sec3.6]3.6 and [Sec sec3.7]3.7 and include extremely improbable values for data items or inconsistencies between related data items. Improbable signal-to-noise ratios [〈*I*/σ(*I*)〉], swapped *R* values and discrepancies between Matthews coefficient (Matthews, 1968 ▶; Kantardjieff & Rupp, 2003 ▶) and solvent content are just a few examples. Missing important data items also fall into this nuisance error category. Internal data-consistency checks, most of which are already provided in validation reports, should flag such obvious errors and omissions, and authors as well as journal editors and reviewers must be encouraged to take such error reports seriously. While these types of errors do not necessarily invalidate the respective structural models, they do impede data mining.

#### 2.1.2. Non-parsimonious models   

The principle of parsimony or the ‘law of succinctness’ commends selecting a model or hypothesis that postulates as few parameters as possible, thereby eliminating variables that make no significant difference in the observable predictions of the explanatory hypothesis or model. This basic idea dates back to the English logician and Franciscan friar William of Ockham (1288–1347; Occam in Latin spelling) and is known as Occam’s razor: *Numquam ponenda est pluralitas sine necessitate*, or *Multitude must never be proposed without necessity*. In formal terms, it can be formally derived *via* Bayes’ theorem introduced in §[Sec sec2]2: a hypothesis with fewer adjustable parameters will automatically have an increased posterior probability (Jefferys & Berger, 1991 ▶).

In principle, any crystal structure can be presented in space group *P*1 (that is, devoid of any symmetry beyond lattice translations). However, particularly in the cases of a low data-to-parameter ratio that often plague macromolecular structure refinement, the resulting model contains more molecules in the asymmetric unit than is necessary and is therefore not parsimonious. The resulting overparameterization (particularly if the use of NCS restraints is also neglected in such cases) leads to models that may not be completely wrong, yet are invariably of lower quality despite very good statistics. Practically all data-collection programs contain modules that search for appropriate higher symmetry in the data, and even the simple – but often ignored – methods of native Patterson and self-rotation Patterson function analysis (as discussed, for example, by Rupp, 2009 ▶) will almost always reveal the presence of local symmetry compatible with higher space-group symmetry. In such a case, a combination of automated checks and better training of the individuals determining the structures will reduce errors in space-group assignment, as further explained in §[Sec sec3.4]3.4.

The introduction of unwarranted complexity is also exemplified by the PDB entries discussed in §[Sec sec3.4]3.4 and mostly affects overenthusiastically parameterized low-resolution structures. A well publicized case is the initial structure of an ABC transporter, which was built (or forced, one might say) into the wrong-handed electron-density map and then subjected to multi-conformer refinement. Fortunately, the structure was subsequently re-determined (Dawson & Locher, 2006 ▶; Matthews, 2007 ▶), and the original deposition and the *Science* paper describing the incorrect structure were retracted. Such cases of low-resolution data or poor data-to-parameter ratio are particularly conducive to the temptation to find what one seeks, and probably only better training and better supervision can minimize the danger of falling into this type of trap. Overenthusiastic solvent modeling (§[Sec sec3.6]3.6) also generates non-parsimonious models.

#### 2.1.3. Ignoring the evidence   

It is somewhat puzzling that many errors in electron-density interpretation are neither subtle nor within justifiable differences of interpretation. Sometimes, an almost complete absence of evidence or the outright ignorance of contrary evidence seems to be at the origin of model features that are considered to be real. Clearly, such mistakes can almost always be avoided by objectively considering the primary evidence of electron density (our first Bayes term) and keeping Bacon’s warning regarding wishful thinking in mind. In addition, the absence of restraining electron density tends to generate the freedom to violate the second Bayes term: for example, loops (or ligands) built into non-existing electron density almost always provide a source of persistent geometry violations.

Suitable examples are given in §[Sec sec3.1]3.1, where the problem results from the ignorance of electron-density evidence and unjustified overreliance on the refinement programs, which even at atomic resolution (better than 1 Å) cannot escape local minima. An example of the misinterpretation of electron density at atomic resolution was identified (Kantardjieff *et al.*, 2002 ▶) in the case of a concanavalin A model where solvent water molecules were placed into atomic resolution electron density of MPD.

Disregarding a clear absence of evidence, perhaps as a result of expectation bias, seems to be the reason for numerous examples of ligands placed into almost vacuous electron density (Pozharski *et al.*, 2013 ▶). Training in electron-density interpretation and the utilization of tools such as real-space correlation coefficients (Brändén & Jones, 1990 ▶; Kleywegt *et al.*, 2004 ▶; Rupp, 2006 ▶) can prevent at least capital mistakes.

#### 2.1.4. Ignoring prior probability   

The second term in Bayes’ theorem is simply an estimate of the plausibility of the model given solely the prior knowledge that one already possesses. A significant number of problems can be traced to apparent leniency towards implausible features in stereochemistry, in the chemical environment, or in coordination geometry, often combined with improbable refinement results such as absurdly high or low *B* factors. A ‘6σ’ event, which for particle physicists signifies an entirely unexpected and paradigm-changing result (the probability that an event drawn from a normally distributed sample will lie outside of six standard deviations from the mean is only one in 506 797 345) seems to be of little concern to some macromolecular crystallographers as regards acceptance of model errors. Multiple subsections in §[Sec sec3]3 present examples belonging to this category. In this situation, simply paying attention to the anomalies and correcting the model (or, in unresolvable cases, omitting these parts instead of publishing an almost 100% improbable model) should rectify most of the affected model entries.

It has been pointed out to us that a possible reason for the disinclination of crystallographers to be alarmed by ‘6σ’ events could be the practice of expressing electron-density levels as non-normalized σ levels above the mean density. Therefore, high σ levels in 2*mF*_o_ − *DF*_c_ maps or in positive OMIT difference maps are generally desirable. Expressing electron-density levels in actual density units (electrons Å^−3^) might steer away from mental acceptance of high σ levels as universally virtuous. Similarly, normalized variance measures such as real-space *R*-value *Z*-scores (RSRZ scores; Kleywegt *et al.*, 2004 ▶), real-space difference density *Z*-scores (RSZD score) or quantile–quantile (Q–Q) difference plots (Tickle, 2012 ▶) with defined significance levels have been introduced and are provided in PDB validation reports.

## 3. Examples of various misrepresentations and errors in published structures   

### 3.1. General negligence   

A number of approaches can be used to validate the models of X-ray structures, including the most obvious one of looking at the model coordinates displayed together with the corresponding electron-density maps. However, if none of them is utilized properly, or if validation is neglected altogether, incorrect models result.

**Figure 1:**
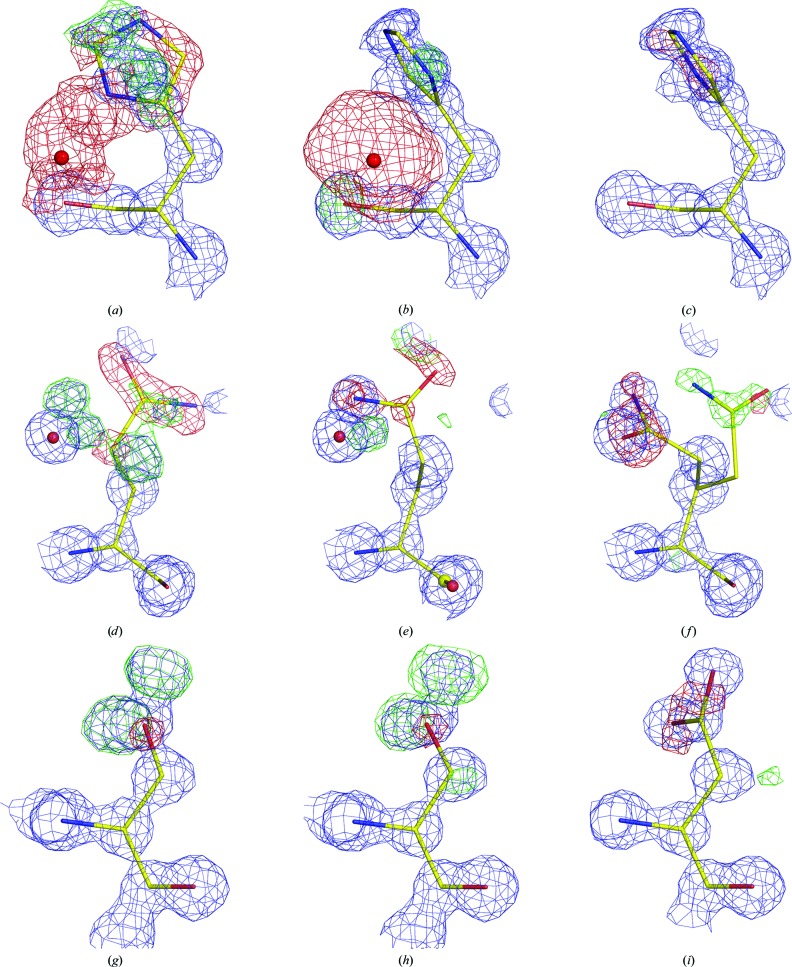
Selected residues from the structure of proteinase K (PDB entry 3i34) accompanied by electron-density maps obtained from the Uppsala Electron Density Server (EDS) (*a*, *d*, *g*), the *PDB_REDO* server (*b*, *e*, *h*) and after manual rebuilding and refinement of the model by the present authors (*c*, *f*, *i*). The 2*mF*_o_ − *DF*_c_ map (blue) is contoured at 1.5σ and the *mF*_o_ − *DF*_c_ map at ±2.0σ (positive contours, green; negative contours, red). (*a*, *b*, *c*) His69 and a spurious Hg ion; (*d*, *e*, *f*) Gln54 with the maps in (*f*) calculated prior to the introduction of the second conformation of this residue; (*g*, *h*, *i*) Asp207, which is represented as Ser207 in the deposited structure. The purple and red spheres represent dubious Hg and water sites included in the original and the *PDB_REDO* models, respectively.

The structure with PDB code 3i34 (Pechkova *et al.*, 2009 ▶) is one in a long series of depositions (PDB entries 3i2y, 3i30, 3i34, 3i37, 3dw1, 3dw3, 3dwe, 3dvq, 3dvr, 3dvs, 3d9q, 3de0, 3de1, 3de2, 3de4, 3de5, 3de6, 3de7, 3ddz and 4dj5) resulting from high-resolution studies of radiation-damage effects on crystals of proteinase K. When the coordinates and electron-density maps downloaded from the Uppsala Electron Density Server (EDS; Kleywegt *et al.*, 2004 ▶) are displayed, it is easy to see that more than 20 side chains are placed out of the otherwise very clear electron density calculated at the atomic resolution of 1.0 Å. The examples illustrated in Fig. 1 ▶ show three residues in the original model of 3i34 (Figs. 1 ▶ *a*, 1 ▶ *d* and 1 ▶ *g*), the model taken from the *PDB_REDO* (Joosten *et al.*, 2009 ▶, 2012 ▶) server (this is a server that automatically re-refines all PDB structures using the latest state-of-the-art tools; Figs. 1 ▶ *b*, 1 ▶ *e* and 1 ▶ *h*) and the same residues after manual rebuilding and refinement (Figs. 1 ▶ *c*, 1 ▶ *f* and 1 ▶ *i*). The wrong rotamer of His69 was originally accompanied by the Hg ion having a *B* factor of 200 Å^2^ (Fig. 1 ▶ *a*). After *PDB_REDO* this residue fits the electron density better, but the rotamer seems to be opposite and the *B* factor of the Hg ion refined to 111 Å^2^ (Fig. 1 ▶ *b*). After manual reconstruction the features in the difference maps disappeared almost completely (Fig. 1 ▶ *c*). Similarly, the rotamer of Gln54 (Fig. 1 ▶ *d*) was somewhat improved by *PDB_REDO* (Fig. 1 ▶ *e*), but in fact manual rebuilding was necessary to move it to its major conformation (Fig. 1 ▶ *f*) and to reveal that the original rotamer corresponded to a low-occupancy secondary conformation. The residue 207 modeled as serine (Figs. 1 ▶ *g* and 1 ▶ *h*) is in fact an aspartic acid (Fig. 1 ▶ *i*), as in the majority of proteinase K structures. The same type of error is present in all of the other structures of proteinase K listed above that resulted from investigations by the same authors. All those models were derived from the early 2.4 Å resolution structure of the Hg complex of protein­ase K (PDB entry 1ptk; Müller & Saenger, 1993 ▶), but were apparently refined automatically without visually inspecting the model and maps. The resulting and persisting model errors demonstrate that even modern remediation and refinement methods, such as those employed in the *PDB_REDO* procedure, might not be capable of automatically moving a side chain to its respective electron density if the starting position is outside the radius of convergence of a method, punctuating the notion that verification by the experimenter (and manual correction) is absolutely necessary.

**Figure 2:**
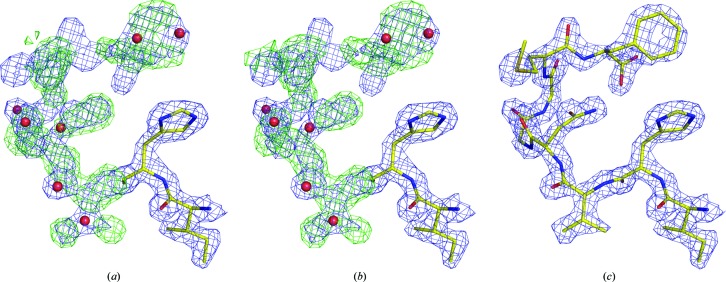
C-terminal fragment of the *B* chain from PDB entry 2p68 with 2*mF*_o_ − *DF*_c_ (blue at +1.5σ) and *mF*_o_ − *DF*_c_ (green at +2.0σ) maps obtained from (*a*) the EDS, (*b*) the *PDB_REDO* server and (*c*) after insertion and refinement (by the present authors) of six residues missing from the original model. Red spheres mark incorrectly placed water molecules in the original and the *PDB_REDO* models.

The structure with PDB code 2p68 (RIKEN Structural Genomics/Proteomics Initiative, unpublished work) contains two chains of the same protein molecule related by noncrystallographic symmetry. In the deposited model, the *A* chain consists of 248 residues, all occupying very clear electron density, but chain *B* contains only 242 residues. Nevertheless, there is excellent electron density for the last six residues of chain *B* that are missing from the deposited model, whereas some water molecules are scattered in this density (Fig. 2 ▶ *a*). The automatic *PDB_REDO* procedure was not able to rectify this situation (Fig. 2 ▶ *b*); only manual insertion of the missing residues satisfied the electron-density map around this fragment (Fig. 2 ▶ *c*). In this case, the missing part could have simply been copied from the other chain and verified by inspection.

**Figure 3:**
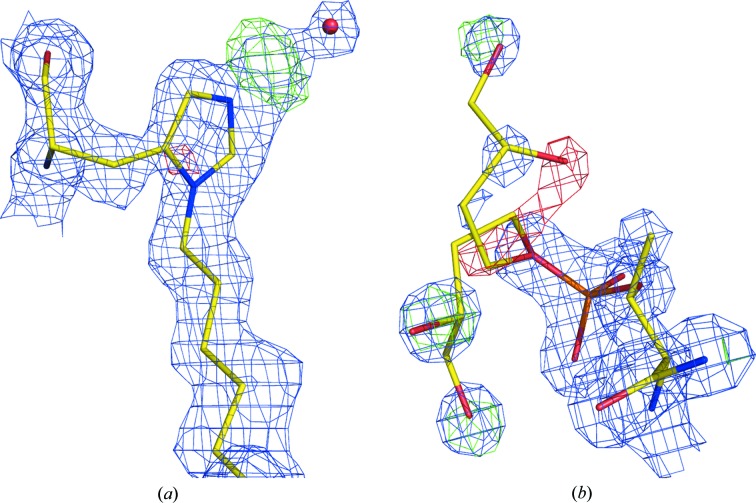
Two fragments of an inhibitor binding to the Polo-box domain of Plk1 (PDB entry 4mlu). (*a*) The environment of a histidine moiety of the ligand superimposed on the 2*mF*_o_ − *DF*_c_ map (blue at +1.0σ) and the *mF*_o_ − *DF*_c_ map at ±3.0σ (positive contours, green; negative contours, red), strongly suggesting the existence of a substituent at the N^∊^ atom and beyond. (*b*) The environment of a phosphate group at the other end of the ligand with the 2*mF*_o_ − *DF*_c_ map (blue at +1.5σ) and the *mF*_o_ − *DF*_c_ map at ±3.0σ (positive contours, green; negative contours, red), suggesting that just a few water molecules are present rather than the two conformations of the expected phosphoester moiety.

An inhibitor is very clearly present in the high-resolution (1.45 Å) structure of its complex with the Polo-box domain of Plk1 (PDB entry 4mlu; Qian *et al.*, 2013 ▶), but in an attempt to fit the expected chemical entity to the map the authors disregarded very clear electron density that indicates a covalent modification of the N^∊^ atom of a histidine moiety (Fig. 3 ▶ *a*). Conversely, at the other end of this inhibitor the authors fitted the expected phosphate-bound chain into density that can only accommodate well ordered water molecules. This was performed by assuming twofold disorder of the chain (Fig. 3 ▶ *b*)

A fairly typical example of the inconsistencies found within a publication, or between a publication and the corresponding PDB deposition, is provided by a neutron structure of phosphate-free RNase A (PDB entry 3a1r; Yagi *et al.*, 2009 ▶). According to Table 1 in the publication, the diffraction data were collected to a resolution of 1.4 Å and all of these data were used in refinement, although the effective resolution was defined as 1.7 Å. The latter value is given as the resolution in the PDB deposition without any clarifying remarks, whereas the outermost shell is defined there as 1.49–1.40 Å. Although the total number of unique reflections is the same in the PDB deposition and in the publication, the completeness of the outermost shell is given as 23.7% in the former and 19.5% in the latter. The *R*_merge_ for all data is given as 7.1% in Table 1 and 8.6% in the text of the paper, both for the same number (31 649) of observed reflections (with all corresponding records in the PDB deposition marked NULL). Although these discrepancies and omissions cannot be characterized as major errors, the resulting inconsistencies clearly impede data mining and serve as a reminder that consistency between publication and deposition should be assured with care.

### 3.2. Improbable numbers   

Several recent papers (Karplus & Diederichs, 2012 ▶; Diederichs & Karplus, 2013 ▶; Evans & Murshudov, 2013 ▶; Luo *et al.*, 2014 ▶) have discussed the estimation of the resolution cutoff for diffraction data and have advocated its extension beyond the customary limit [often set to guarantee a 〈*I*/σ(*I*)〉 of at least 2.0 in the highest resolution shell]. We have inspected the distribution of the high-resolution 〈*I*/σ(*I*)〉 values reported in PDB depositions and have realized that some of them are, with high probability, misrepresented or not valid.

Of the 80 939 X-ray structures in the PDB (27 June 2013), 49 589 report a numerical value for the high-resolution 〈*I*/σ(*I*)〉 (in the remaining entries, NULL is quoted). The distribution of the high-resolution 〈*I*/σ(*I*)〉 values is presented in Table 1 ▶. A considerable fraction of the structures report highly improbable values of this parameter. The most extreme are PDB entry 2ie1 (Ohishi *et al.*, 2008 ▶), with 〈*I*/σ(*I*)〉 = 6400 for both the overall and highest resolution ranges, and PDB entry 1iux (Gatti *et al.*, 1996 ▶), reporting 〈*I*/σ(*I*)〉 = 0.01 for the highest resolution range in the PDB deposition (but 1.95 in the publication). These two examples testify to a degree of carelessness in depositing the results in the PDB, but Table 1 ▶ also shows that in a large percentage of the investigations the full diffraction potential of the crystals was not utilized, since in about 50% of the depositions the highest resolution 〈*I*/σ(*I*)〉 exceeds 3.0.

There are many examples of inconsistencies between the numerical values provided by depositors and the actual features of their structure models. In 2168 PDB entries the solvent content (*V*_S_) differs by more than 10% from the provided value (*V*_M_) of the Matthews coefficient [*V*_S_ = 100% × (1 − 1.23/*V*_M_); Matthews, 1968 ▶]. In 94 cases, the solvent content is given as a fraction instead of a percentage. 33 depositions declare a *V*_M_ of smaller than 1.23 Å^3^ Da^−1^ and 41 give a *V*_M_ of greater than 10.0 Å^3^ Da^−1^, both highly improbable values for other than very special cases (Kantardjieff & Rupp, 2003 ▶). In one case (PDB entry 2ymy; Makbul *et al.*, 2013 ▶) both *V*_M_ and *V*_S_ have the same numerical value of 99, which, coincidentally, fulfils the Matthews formula.

There are 89 structures in the PDB with *R*_free_ < *R*. The largest difference is in PDB entry 2rtn at 1.34 Å resolution (Katz, 1997 ▶), with the *R*/*R*_free_ values reported in the PDB as 27.6/20.0%; however, in the corresponding publication only the *R* value is given as 19.5%, indicating a possible swap of these two values.

The very large structure 2qzv of the vault shell (Anderson *et al.*, 2007 ▶) was elucidated by cryo-electron microscopy and was extended to 9 Å resolution by X-ray crystallography. The reported *R* factor is 61.5%, *i.e.* higher than the value of 59% characterizing a random acentric set of atoms (Wilson, 1950 ▶). The cell dimensions in the PDB file have exceedingly high precision, with *a* = 631.449, *b* = 464.724, *c* = 584.572 Å, β = 123.84°, whereas the cell dimension of halite (NaCl), used as a standard, is known with fewer than five significant digits as 5.6400 (5) Å (Barrett & Wallace, 1953 ▶).

Wilson *B* values are reported as negative for 32 PDB entries and as exactly zero for another 70 depositions, whereas there are ‘only’ three cases of negative values and seven of exactly zero mean *B* values for all atoms. The deposition 3q2q (Wallrapp *et al.*, 2013 ▶) reports a Wilson *B* value of 37 374 Å^2^ and has a mean atomic *B* of 51 Å^2^.

In the structure 3ifx, individual *B* factors were evidently refined at 3.56 Å resolution, contradicting the claim in the publication (Cieslak *et al.*, 2010 ▶) that group *B*-factor refinement was utilized. There are 27 atoms with *B* = 2.0 Å^2^ and 35 atoms with *B* = 500 Å^2^. In the model entry, the average *B* value is listed as 117.5 Å^2^ for 2780 protein atom sites, but the publication reports 66.4 Å^2^ for 2818 atom sites.

Most of these errors and mistakes could have been eliminated through careful scrutiny of numerical values upon submission to the PDB and making sure that the values in the PDB file correspond to those in the publication. In addition, related implausible numerical values could readily be intercepted by automated validation programs during deposition and during further database-remediation cycles.

There are several investigations of protein crystal structures by the powder diffraction method. In many of them the numerical values of the wavelength and cell dimensions are given with excessive and unrealistic precision. For the structure 1ja2 (Von Dreele, 2001 ▶) of hen egg-white lysozyme (HEWL) the *a* unit-cell parameter is given as 79.1317 (11) Å, with a relative precision of 1.4 × 10^−4^, whereas the unit-cell volume (a derived value) of 238 135 (8) Å^3^ has a relative precision of 3.4 × 10^−5^. For the structure of HEWL with 10.0 m*M* gadolinium salt at pH 3.5 (Wright *et al.*, 2008 ▶), the estimated value of the wavelength is 1.54999 (3) Å, with a relative precision of 2.0 × 10^−5^, but the unit-cell parameter *a* = 79.119566 (20) Å has an implausible precision of 2.5 × 10^−7^. By comparison, the unit-cell parameter of a silicon crystal, which was used for calibration purposes, provided by the National Institute of Standards and Technology (NIST) is 5.43123 (8) Å and has relative accuracy of 1.5 × 10^−5^; thus, the HEWL unit-cell parameters would be almost two orders of magnitude more accurate than the world best standard of crystal cell dimensions. This unrealistic error estimation often results from the fact that computer programs provide information about statistical error, but not total error, which is a combination of statistical and systematic errors.

A type of error presumably resulting from the ‘copy-and-paste’ practice is exemplified by the PDB depositions 1gmu and 1gmw (Song *et al.*, 2001 ▶), in which the r.m.s. deviations of bond lengths and angles from ideality are quoted as 0.004631 Å and 1.30162° (identical for both structures), *i.e.* with a precision corresponding to 1/10th of the particle size of an electron.

The presence of ANISOU records in a PDB coordinate file may result from anisotropic refinement of the atomic displace­ment parameters (ADPs) of individual atoms or from translation/libration/screw (TLS) treatment of rigid-body fragments. Table 2 ▶ shows, split into resolution ranges, the number of depositions with ANISOU records and the number of depositions declared in the file header as refined by the TLS approach. The difference between these two numbers may identify structures refined anisotropically (especially at high resolution) or those refined with TLS parameterization but not declared as such (especially at low resolution). Thus, the double meaning of the ANISOU records in PDB files introduces significant confusion in the description of the refinement method if the depositors neglect to provide full information about the refinement procedure. It would be beneficial to differentiate the two cases by using different record types, for example ANISOU and TLSU. Taken at their face value, the data in Table 2 ▶ may indicate that a significant number of structures at resolutions lower than 1.5 Å (probably the lowest resolution at which anisotropic refinement could be justified, although barely) were indeed refined anisotropically, with fewer actual observations than refined parameters.

### 3.3. Blindly following program defaults   

Crystallographic software has been developed to a point where it is often possible to simply use the default values of the parameters in data processing, structure determination or structure refinement. However, the use of defaults under all circumstances may be quite counterproductive. An example is provided by the structure of a DNA hexanucleotide refined at 3 Å resolution (PDB entry 3ulm; Mandal *et al.*, 2012 ▶). The small size of the unit cell and the limited data resolution resulted in only 147 measured reflections. Nevertheless, the authors refined the structure with a free *R* factor (which claimed 6.9% of the data, or ten reflections) and presented the results in 20 resolution shells, of which at least half must have contained none of these test reflections. Since the refinement also included individual *B* factors, there were clearly not sufficient data for a statistically valid refinement procedure.

### 3.4. Symmetry   

Every crystal structure (except those in space group *P*1) can be expressed in some lower symmetry subgroup. However, presenting a higher symmetry structure in lower symmetry involves the introduction of unnecessary, redundant parameters, violating the principle of parsimony (§[Sec sec2.1.2]2.1.2). In such cases, many refined parameters are highly correlated and the refinement process may be severely biased as a result of the presence of multiple symmetry-equivalent atoms. In addition, equivalent diffraction data are treated separately, thus preventing the possibility of improving their quality by averaging. In complicated pseudo-symmetric cases the decision about the proper space group may be difficult (Thompson & Yeates, 2014 ▶), but sometimes clearly high-symmetry structures are published and deposited in an erroneously assumed lower symmetry representation. This situation is also well known in small-molecule crystallography, where it has been the subject of a crusade by Richard Marsh (*e.g.* Herbstein & Marsh, 1982 ▶; Marsh & Bernal, 1995 ▶). For more details, see also the presentation by Ton Spek (http://www.cryst.chem.uu.nl/spek/ppp/spek_marsh.ppt).

The structure 3jtt (Liu *et al.*, 2011 ▶) presents three major histocompatibility complex (MHC) molecules in the asymmetric unit in the orthorhombic space group *P*2_1_2_1_2_1_, with *a* = 128.99, *b* = 129.01, *c* = 129.03 Å, refined to *R* = 21.4% and *R*_free_ = 25.5%. Superposition of these three individual complexes gives an r.m.s.d. for the C^α^ atoms of 0.143, 0.156 and 0.173 Å, *i.e.* much smaller than the declared maximum-likelihood coordinate error of 0.4 Å. Indeed, the diffraction data can be merged with an *R*_merge_ of 2.8% and the structure can be easily refined (without additional rebuilding) in space group *P*2_1_3 to *R* = 17.3%. Even a superficial glance at the result shows a perfect trimer positioned around the threefold axis along the space diagonal of the cubic unit cell.

Three related crystal structures, 2pp0, 2pp1 and 2pp3 (Yew *et al.*, 2007 ▶), have very similar unit-cell dimensions. Two of them are presented in space group *P*422 with three independent molecules, but one (2pp1) is in the orthorhombic space group *P*222, with *a* = 123.187, *b* = 173.826, *c* = 173.792 Å and six unique molecules in the asymmetric unit. Three molecules (*A*, *B*, *C*) in 2pp1 can be superposed on the other three (*D*, *E*, *F*) and the r.m.s.d. for all 3 × 394 C^α^ atoms is 0.63 Å, whereas the declared accuracy of the coordinates is ∼0.3 Å. The diffraction data for entry 2pp1 merge in 422 symmetry with an *R*_merge_ of 5.2% and the structure refines in space group *P*422 to an *R*/*R*_free_ of 19.7/25.3%, while the *R* factor reported for space group *P*222 is 22.3% with a rather similar *R*_free_ of 23.7%. These crystal structures actually consist of two perpendicular octamers, one generated from molecule *A* and centered around the 422 site at 0, 0, ½ and the other generated from molecules *B* and *C* around the 222 site at 0, ½, 0. Interestingly, superposition of these two octamers (8 × 394 C^α^ atoms each) gives an r.m.s.d. of only 0.37 Å for the structure 2pp0, a value comparable to the accuracy of the coordinates.

Two PDB depositions, 1zwk and 1zwl (Gorman & Shapiro, 2005 ▶), describe the structures of the WrbA protein in apo and FMN-complexed forms. Their cell dimensions are very similar, but the former structure is described in space group *P*222 (with *a* = 73.314, *b* = 73.331 Å), whereas the latter structure assumed space group *P*4_2_22. The two molecules in 1zwk superpose their 165 C^α^ atoms with an r.m.s.d. of 0.33 Å, whereas the declared coordinate accuracy based on *R*_free_ is 0.32 Å. Indeed, these two ‘independent’ molecules are related by a perfect twofold axis parallel to the [110] direction, and the diffraction data merge in 422 tetragonal symmetry with an *R*_merge_ of 2.7%. It is possible to easily refine the 1zwk structure in space group *P*4_2_22 to an *R*/*R*_free_ of 18.9/27.3%, whereas the deposited information for *P*222 cites 22.9/27.3%.

The structure 2a8y (Zhang *et al.*, 2006 ▶) is presented in space group *P*1 with 12 molecules in a unit cell that is very nearly rhombohedral in shape. This was noticed by the authors, who wrote

The reflection images were first indexed and processed in a primitive rhombohedral lattice with *a* = 138.97 Å and *c* = 163.33 Å. Subsequent merging of the data with either rhombohedral Laue symmetry failed to give acceptable *R*_sym_ values. Indexing and processing the data set in lattices with lower symmetry, including *C*-centered orthorhombic, primitive monoclinic, and *C*-centered monoclinic were also unsuccessful. Finally the data set was processed and merged in the triclinic space group with approximate unit-cell dimensions of *a* = 96.60 Å, *b* = 96.56 Å, *c* = 96.63 Å, α = 91.57°, β = 91.23° and γ = 91.52°.

It was overlooked, however, that the rhombohedral cell can be expressed as a *C*-centered monoclinic lattice in three different ways, and that the autoindexing program lists only one of the possibilities. In fact, this structure has *C*2 symmetry, confirmed by *R*_merge_ = 4.6%, and can be refined with statistics comparable to the results obtained in *P*1. This case suggests that it would be useful if all data-processing programs took into account all possible supergroup/subgroup relations during the indexing and merging procedures and presented the suggestions to the users, analogously to what is already implemented in programs such as *XPREP* (Sheldrick, 2003 ▶), *POINTLESS* (Evans, 2006 ▶, 2011 ▶) or *phenix.xtriage* (Zwart *et al.*, 2005 ▶).

**Figure 4:**
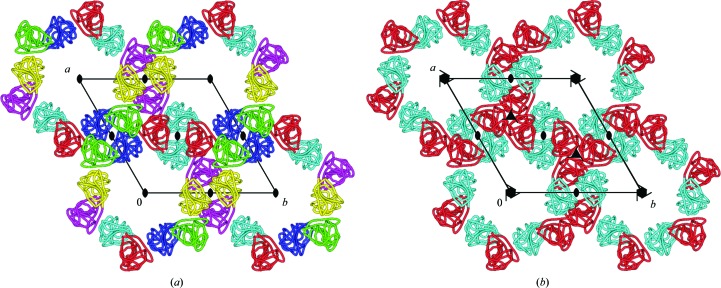
Packing of the molecules in PDB entry 2w9s. Shown are the unit cell and symmetry elements of the original space group *P*2 (*a*) and of the true space group *P*6_2_ (*b*). Molecules that are equivalent by space-group symmetry elements are presented in the same color.

Six molecules are present in the asymmetric unit of space group *P*2 in the crystal structure of S1:DHFR (PDB entry 2w9s; Heaslet *et al.*, 2009 ▶). The deposited cell dimensions are *a* = 115.242, *b* = 67.375, *c* = 115.249 Å, β = 120.00°, and the *R*/*R*_free_ values are 20.2/23.7%. However, the diffraction data can be merged in space group *P*6_2_ with an *R*_merge_ of 5.8% and the model, now consisting of only two independent molecules, can be refined to an *R*/*R*_free_ of 19.9/23.9%. Inspection of the packing of the molecules in the unit cell, shown in Fig. 4 ▶, strongly suggests the hexagonal symmetry of this structure.

**Figure 5:**
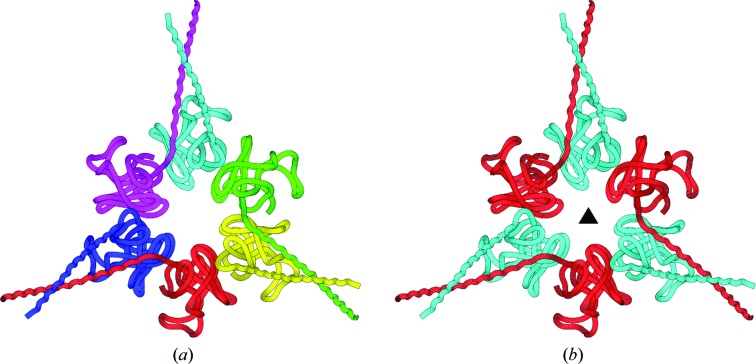
The hexamer of protein molecules in PDB entry 3m9b, originally presented in *P*2_1_ symmetry (*a*), are in fact placed around the space-diagonal threefold axis of the true space group *P*2_1_3 (*b*). Symmetry-equivalent molecules are shown in the same color.

The structure of the proteasomal ATPase Mpa (PDB entry 3m9b; Wang *et al.*, 2010 ▶) is presented at 3.94 Å resolution in space group *P*2_1_, with unit-cell dimensions *a* = 176.787, *b* = 176.652, *c* = 176.633 Å, β = 90.04° and 12 independent molecules in the asymmetric unit. In fact, the diffraction intensities merge very well when cubic symmetry is applied, with an *R*_merge_ of 3.0%. The structure refines in space group *P*2_1_3 to an *R*/*R*_free_ of 25.1/28.0%, while the deposited values for the *P*2_1_ space group are 27.6/30.4%. The structure consists of unique dimers arranged around the threefold axis to form symmetric hexamers (Fig. 5 ▶).

One can argue that errors in symmetry determination are not critical for the interpretation of structure–function relationships or for drug-discovery studies. However, sometimes wrong symmetry can create controversy that puts drug discovery on hold or pushes it in the wrong direction. The structure 1lox of 15S-lipoxygenase with an inhibitor solved in space group *R*32 (Gillmor *et al.*, 1997 ▶) was followed by the structure 2p0m derived from reinterpretation of the structure factors in terms of perfect twinning in space group *R*3 (Choi *et al.*, 2008 ▶). The shape and size of the substrate-binding cavity of the reinterpreted model is significantly changed and the cavity is no longer able to accommodate the ligands proposed to bind in this pocket. If the original diffraction data had not been available, it would not have been be possible to resolve this controversy and the drug-discovery community would have no way of knowing the correct interpretation.

The decision about which crystal symmetry to select should not be based exclusively on the appearance of cell dimensions (metric symmetry) obtained from the initial indexing of the diffraction pattern. More important is the agreement of reflection intensities with the proposed point-group symmetry, but even this may not be decisive enough in cases of merohedral twinning. However, if the unit-cell parameters suggest the possibility of higher symmetry, it should be convincingly disproved before selecting lower symmetry, especially if the lower symmetry space group is rare, such as *P*2 or *P*222 (Wukovitz & Yeates, 1995 ▶). Although in some cases peculiar­ities such as pseudomerohedral twinning make proper selection of the space group difficult, none of the examples given above show any indication of such problems.

Several examples discussed in this section illustrate the problem of accuracy *versus* precision of unit-cell parameters. Following the format requirement adopted by the PDB, the majority of depositions give cell dimensions with a precision of three decimal digits (*i.e.* to within 0.001 Å). The indexing programs usually print these values with at least such a precision, even if the unit-cell parameters are of the order of several hundred angstroms, and these values are copied in all successive stages of structure determination up to PDB submission. The maximum achievable experimental *accuracy* of wavelength determination and crystal-to-detector distance setting cannot, for practical reasons, reach the *precision* of six meaningful digits and thus the presentation of unit-cell parameters with such high precision borders on the worship of numerology. Comparison of the unit-cell parameters quoted above for crystal structures presented in artificially lower symmetry shows that parameters that are truly identical by symmetry may differ by more than 0.1 Å. This suggests that macromolecular crystallographers should be more realistic in the estimation of the precision of their unit-cell parameters.

### 3.5. Confusing placement of molecules   

**Figure 6:**
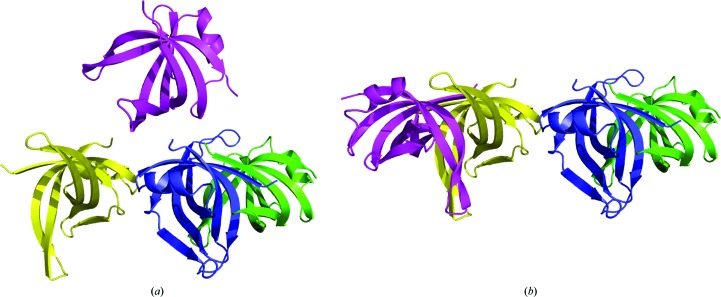
Four protein molecules in the unit cell of PDB entry 1woc. (*a*) Original presentation as one dimer and two monomers; (*b*) after regrouping by an appropriate symmetry operation it is clear that this structure consists of two identical dimers.

Four independent molecules found in the asymmetric unit of a crystal of the protein PriB (PDB entry 1woc; Shioi *et al.*, 2005 ▶) are presented as one dimer and two monomers (Fig. 6 ▶ *a*). However, if one of the two ‘lonely’ monomers is transformed by one of the crystallographic symmetry operations, to­gether they form a second dimer exactly the same as the first one (Fig. 6 ▶ *b*). The originally presented structure is perfectly correct from a strictly crystallographic point of view, but is illogical from the point of view of biology and may severely confuse users of the PDB who are less fluent in symmetry transformations. The optimal arrangement of subunits in multimeric assemblies can be identified by the *PISA* server (Krissinel & Henrick, 2007 ▶).

In the structure 3ozq (Park *et al.*, 2011 ▶) the unique molecule is placed about 12 unit cells away from the origin. This, again, is formally correct from a crystallographic point of view, although it may confound not only PDB users but also some crystallographic programs that are limited to take into account only symmetry transformations in the vicinity of the standard unit cell. In a very large number of other PDB structures the molecules lie outside of the defined unit cell; again, while such a model representation is crystallographically valid, it should be avoided to minimize confusion (Dauter, 2013 ▶).

### 3.6. Chemistry   

**Figure 7:**
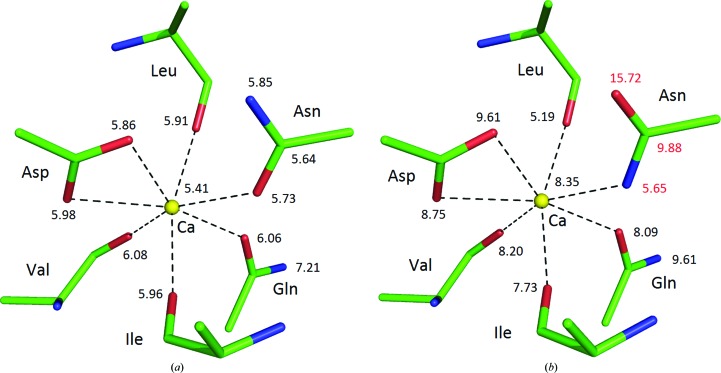
Coordination of the ‘strong’ calcium site (yellow sphere) in savinase with the refined values of atomic displacement parameters (*B* factors; Å^2^) of the relevant atoms: (*a*) in PDB entry 1gci refined at 0.78 Å resolution, (*b*) in PDB entry 1svn refined at 1.4 Å resolution.

All subtilisin-like enzymes contain the so-called ‘strong’ calcium site pseudo-octahedrally coordinated by O atoms derived from three carbonyls, two amides and one carboxyl group (Gilliland & Teplyakov, 2011 ▶; Fig. 7 ▶ *a*). However, among the 15 crystal structure depositions for savinase (subtilisin from *Bacillus lentus*), there are three, 1svn, 1tk2 and 3bx1, in which the amide N atom of asparagine, instead of the O atom, is in the immediate vicinity of the Ca^2+^ ion (Fig. 7 ▶ *b*). Such an arrangement is not possible since an amide N atom with two H atoms is not a ligand for metal coordination. Inspection of the atomic displacement parameters, marked in Fig. 7 ▶, clearly shows that the amide group should be flipped in these structures.

**Figure 8:**
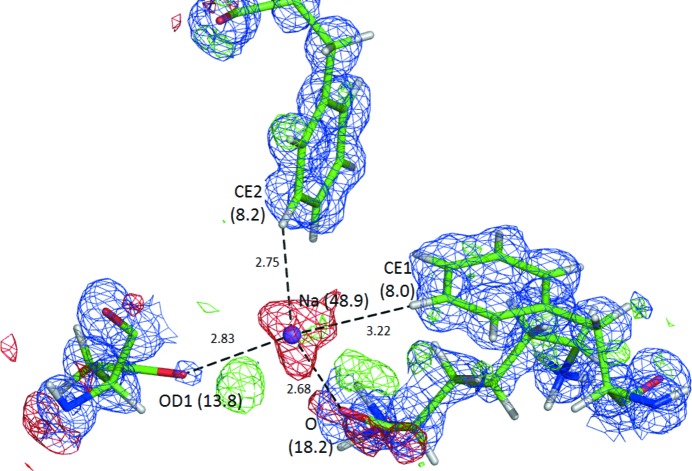
A fragment of PDB structure 3e0k around the site of a rather improbable Na^+^ ion. The *B* factors (Å^2^) of the Na^+^ ion and selected neighboring atoms are shown in parentheses and the distances (in Å) of two close O and two H atoms are also shown. The 2*mF*_o_ − *DF*_c_ map (blue) is contoured at 1.5σ and the *mF*_o_ − *DF*_c_ map at ±2.5σ (positive contours, green; negative contours, red).

Not only are the ligands of metal ions sometimes misinterpreted, but also the metal ions themselves. Fig. 8 ▶ shows the site of a rather improbable Na^+^ ion in the structure 4e0k (Liu *et al.*, 2012 ▶) refined at 0.97 Å resolution. In spite of two favorable contacts with O atoms, features such as the close vicinity of two phenyl rings, negative difference electron density and a much higher *B* factor than for the surrounding atoms all significantly reduce the probability that this site is in reality occupied by a metal ion or, indeed, by any atom.

**Figure 9:**
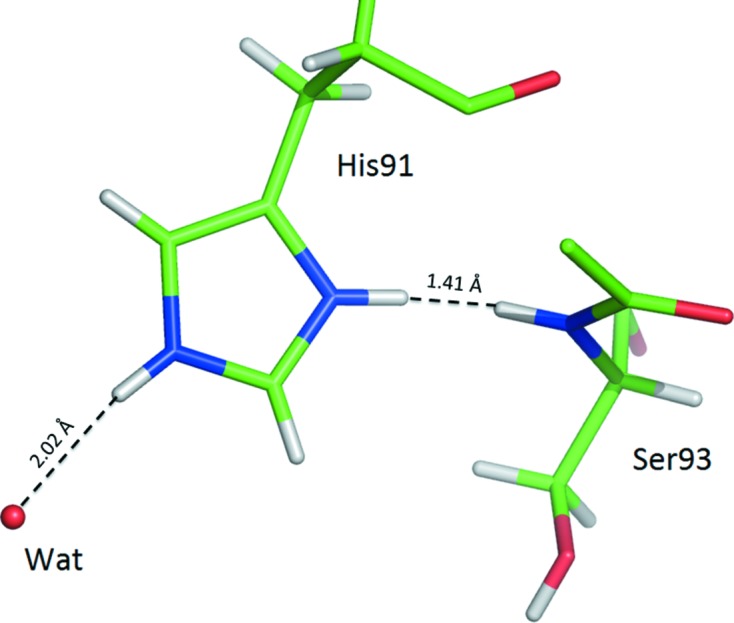
A fragment of bovine trypsin from PDB structure 3unr that includes the residue His91 that is presented as doubly protonated. However, such an assignment of H atoms (gray) is in conflict with the neighboring peptide N—H group of Ser93.

In investigations of the protonation states of carboxylates and histidines (Fisher *et al.*, 2012 ▶), it was concluded that a resolution of 1.2 Å does not provide sufficiently high positional accuracy of atoms to convincingly derive protonation states of the three histidine residues in the structure of bovine trypsin. However, all three histidine residues are doubly protonated in the deposited structural model 3unr (Fisher *et al.*, 2012 ▶), which also contains H atoms. Inspection of the vicinity of the residue His91 shows that its N^δ1^ atom and the peptide N atom of Ser93 form a favorable hydrogen bond at 2.97 Å but, curiously, both N atoms are protonated (Fig. 9 ▶). Since the peptide group undoubtedly has the N—H form, the His91 residue must be neutral, with only one hydrogen attached to its N^∊2^ atom.

More subtle protonation issues often plague ligand proton­ation/tautomeric forms and can be combined with non-conventional atom numbering (Jaskolski, 2013 ▶). An example of incorrect protonation of a ligand (spermine) can even be found in a record-setting ultrahigh-resolution structure of Z-DNA (Brzezinski *et al.*, 2011 ▶).

**Figure 10:**
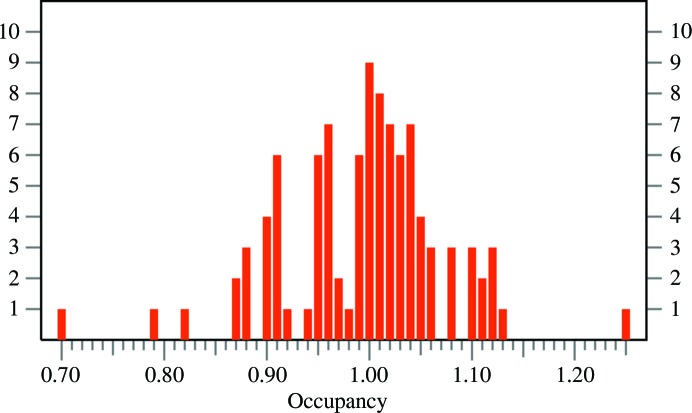
A histogram showing the distribution of sums of site occupancies for individual atoms in double-conformation fragments of the structure of crambin (PDB entry 3nir) refined at the ultrahigh resolution of 0.48 Å.

In the entry 3nir for crambin (Schmidt *et al.*, 2011 ▶), based on diffraction data at the record high resolution of 0.48 Å, the occupancies of atoms in fragments with multiple conformations were refined individually for each site. As a result, the sum of occupancies of 99 pairs of sites belonging to the same atom varies between 0.70 and 1.25 (Fig. 10 ▶). Whereas combined occupancies can be occasionally smaller than 1.0, values larger than 1.0 are not only chemically illogical but are physically impossible.

The majority of individual solvent sites in the ordered solvent region around macromolecules are occupied by water molecules, but some of these sites may belong to certain ions present in the crystallization medium. Identification of ions such as NH_4_^+^, Na^+^ and Mg^2+^, all of which are isoelectronic with water (ten electrons), cannot be achieved on the basis of electron density or atomic displacement parameters alone. Identification of such ion sites can only be made by interpretation of their bonding and coordination environment. A useful diagnostic tool for the identification of metal ions has been recently introduced in the form of a web server, *CheckMyMetal* (Zheng, Chordia *et al.*, 2014 ▶). In four structures of RNA polymerase [PDB entries 1iw7 (Vassylyev *et al.*, 2002 ▶), 1smy (Artsimovitch *et al.*, 2004 ▶), 2a68 and 2a69 (Artsimovitch *et al.*, 2005 ▶)] there are 485, 362, 562 and 487 magnesium ions, respectively. The authors indicated in supplementary material that solvent molecules with *B* values less than a certain threshold and *F*_o_ − *F*_c_ electron density over 5σ were simply treated as Mg^2+^ ions. Any chemical interpretations of such features by future users will, however, be at least dubious.

### 3.7. Missing or inconsistent information   

Since the technical description of the structure-determination process is nowadays often published as supplementary material (if at all), such information should be directly available from the PDB entry. However, the headers of many depositions provide only very fragmentary information about the structure-solution process. An extreme example is PDB entry 2hyd (Dawson & Locher, 2006 ▶), which corrected a series of faulty structures withdrawn from the PDB, in which the header contains ‘NULL’ for almost all of the fields that describe how the structure was determined.

A requirement that PDB depositions of crystallographic models must be accompanied by the experimental diffraction intensities or structure factors (SFs) from which the atomic parameters have been derived has been in force since February 2008. While this requirement is now generally enforced by the majority of scientific journals and by the PDB, it is not obvious that the parameters describing the deposited experimental data are always correct or, in the worst cases, that the model was refined against the correct version of the data set. A superficial analysis of PDB depositions with SF data, using the 〈*I*/σ(*I*)〉 statistics as a criterion, shows that in 10% of the cases the user-reported 〈*I*/σ(*I*)〉 differs from the value calculated from the data. In many cases, there is a strong indication that the refinement protocol was performed with a different version of the SF file than the one that was ultimately deposited. In situations with twinned data, where the refinement program ‘updates’ the experimental data with twin corrections, this could lead to potentially serious problems.

## 4. Structural genomics activities   

PDB depositions from structural genomics (SG) programs worldwide deserve special consideration. Structures determined by SG programs constitute 14% of the entire PDB and 25% of non-redundant depositions. However, almost 80% have not been described in peer-reviewed journals and, taking into account the downscaling of many SG programs, may never be published. For this reason, the quality and complete­ness of a deposition becomes even more important, as the evaluation of a structure in such cases is based entirely on the information in the PDB. For example, when structure determination is not described in a subsequent publication, the title of the PDB deposition is often critical to determine whether the deposition is relevant to a particular data-mining search. The title ‘protein of unknown function’ is useless, requiring one to infer indirect information from sequence-similarity searches, which is not always straightforward.

By multiple measures, SG centers have produced structures that are, on average, of higher quality than typical PDB depositions. For example, the header of a PDB deposition is used to report numerous parameters describing the data-collection and structure-determination process. When a submitter does not report a particular parameter in a PDB deposition, it is set to ‘NULL’ in the header, and many depositions have a significant number of parameters with ‘NULL’ values. The average number of ‘NULL’ parameters is lower for depositions coming from SG centers (Domagalski *et al.*, 2014 ▶) owing to the better experience of the depositors and the use of sophisticated software tools in the SG centers.

Similarly, the quality of the atomic coordinates in macromolecular structures (as measured by both the *R* value and agreement with known stereochemistry) is also better for SG depositions, even though the SG centers do not have a unified standard for PDB deposition. Especially bothersome is the lack of a single standard for handling regions of weak or absent electron density, where multiple approaches have been used even within a single SG center. Generally, there are two approaches to modeling atoms without sufficient electron density: (i) placing atoms in the most probable places and setting their occupancy to zero, or (ii) removing the undefined atoms from the model altogether, to leave an incomplete or missing chemical moiety. In the latter case, there are multiple options: omit only the atoms without density, omit side chains and retain main-chain atoms in the most probable places, or omit entire residues. There are various arguments that justify the superiority of one approach over another, but when the model is used by programs calculating various macromolecular features, the results obtained may strongly depend on the approach used by the depositor. The inability of SG to establish a single unified validation standard for PDB depositions is one of the major remaining unmet challenges of structural genomics (Chruszcz *et al.*, 2010 ▶). The existence of such a standard would be of value to depositors outside of SG and to the biological community at large.

The requirement that structure factors be included with crystallographic PDB depositions is of great benefit as it allows the re-refinement and re-validation of any recently deposited structure. However, sometimes it is impossible to properly replicate (and possibly correct) the problems of structure solution and refinement without reprocessing the original diffraction images. Several SG centers (JCSG, MCSG, CSGID and SSGCID) have made their diffraction images available to the scientific community. The availability of these ‘raw’ data allows full re-evaluation of the structure and is an excellent source of ‘difficult’ test cases for methods development. In the past, the prohibitive cost of the vast data storage required made such solutions impractical. Today, however, the cost of the hardware needed for storage of all diffraction data is insignificant in comparison to the cost of structure determination, even for a very productive laboratory or SG center. What is expensive is a database that will give seamless access to all diffraction images, including a brief description of the data. It is relatively easy to establish such a database within a single laboratory, but much more difficult to create a database that would work across many laboratories, even within one multi-institution consortium.

## 5. Conclusions   

Almost two decades ago, in a paper appropriately entitled ‘*Braille for Pugilists*’, Kleywegt & Jones (1995 ▶) made the following suggestion:

The person who solves the structure has to be absolutely merciless in judging his own model; the supervisor must be supercritical; even the co-authors should be more critical than the worst nit-picking referee will ever be; the referees should demand to be convinced that the structure is correct; and the editors should start listening to their referees.

Unfortunately, as the presented examples have shown, this sound advice has not always been followed.

In 2008, the Worldwide Protein Data Bank (wwPDB) organization launched several Validation Task Forces, which have worked out recommendations and guidelines for validation standards to be applied to atomic models deposited in the PDB as the results of X-ray crystallographic investigations (Read *et al.*, 2011 ▶), electron microscopy (Henderson *et al.* 2012 ▶) and NMR studies (Montelione *et al.*, 2013 ▶). These recommendations are being implemented by the PDB (Gore *et al.*, 2012 ▶).

However, one of the most important messages that we have tried to convey here is that validation and the engagement of a reasoning brain should be continuous and prudent constituents of the entire structure-determination process, and not viewed as a final, perhaps automated, corrective measure for inattention or wishful thinking. Some errors and misinterpretations in published and deposited crystal structures are probably statistically unavoidable in times of increasing production of results in this field of science, where the buzzword ‘high throughput’ is often interpreted as ‘fast throughput’ instead of ‘high output’. Nonetheless, many errors could have been avoided if more care had been devoted to proper scrutiny and validation of the results that are released into the public domain. In this context, the main responsibility rests with the senior investigators and it is, therefore, of concern that some of the problematic cases discussed above have originated in high-profile laboratories. Mistakenly, deposition in the PDB is sometimes treated as a nuisance required by granting agencies or journal editors, without full awareness of the fact that the structural models in the PDB are not only used by thousands of scientists for follow-up studies but also as the source of primary information for various databases that could be easily contaminated by ‘rotten apples’. The scale of the contamination can be illustrated by the fact that two of the structures described above (1lox and 2p0m) have been downloaded about 34 000 times each.

Public databases and data repositories are critical to create knowledge in various areas of biological research, such as drug discovery, and for the development of new software tools. The ripple effect of suboptimal structures affects not only crystallography but also subsequent analyses and data-mining studies. It is clear that the original authors of deposited structures can and should re-refine and re-deposit their models whenever they find that they can be improved. Moreover, when a structure is re-refined by others, there should be an established procedure for ‘updating’ a given structure, perhaps by giving the authors of the original deposition a fixed time to comment on the changes or corrections that have emerged. All structures that replace previous PDB depositions should include REMARK records with clearly presented reasons for making the previous versions obsolete. The PDB should also make much more extensive use of the CAVEAT record that would alert users to the presence of potentially questionable features in the depositions.

Another conclusion emphasizes the urgent need to provide adequate training to next-generation crystallographers. Such concerns have been voiced on several occasions (Wlodawer *et al.*, 2008 ▶, 2013 ▶; Rupp, 2009 ▶; Pozharski *et al.*, 2013 ▶; Zheng, Hou *et al.*, 2014 ▶) and they are repeated here in the context of the International Year of Crystallography, IYCr2014. Macromolecular crystallography will realize its full potential only if the community is willing to enforce and maintain the highest standards of its methodology.

## Figures and Tables

**Table 1:** Distribution of the highest resolution *I*/(*I*) values beyond selected thresholds among the 49589 depositions reporting this parameter in the PDB as of 27 June 2013

*I*/(*I*) threshold	Number	%
>1000	10	0.02
>100	25	0.05
>10	1935	3.9
>5	9289	18.7
>3	24421	49.2
>2	40676	82.0
2	8913	18.0
1	736	1.5
0.1	18	0.03

**Table 2:** PDB coordinate depositions with ANISOU records and those declared as refined with TLS parameterization, shown in resolution ranges The difference between these two numbers corresponds to structures that were refined with anisotropic ADPs of individual atoms or refined using TLS but are not declared as such in the PDB file.

Resolution range ()	ANISOU	TLS	Difference	All PDB X-ray structures
0.250.50	1	0	1	1
0.500.75	11	0	11	25
0.751.00	338	3	335	359
1.001.25	1427	77	1350	2136
1.251.50	1997	417	1580	3493
1.501.75	2335	1422	913	12642
1.752.00	2536	2275	261	14298
2.002.25	2398	2263	135	18549
2.252.50	1547	1471	76	8799
2.502.75	1352	1290	62	9903
2.753.00	837	789	48	4688
3.003.25	654	611	43	3758
3.253.50	275	257	18	868
3.503.75	161	152	9	734
3.754.00	72	63	9	242
4.004.25	50	41	9	192
4.254.50	23	23	0	54
4.504.75	7	7	0	51
4.755.00	3	2	1	11
5.005.25	3	2	1	26
5.255.50	3	3	0	4
5.505.75	1	1	0	20
5.756.00	2	2	0	5
>6.00	11	10	1	87
				
All	16044	11181	4863	80947
